# Supplementary material for: FoxO1–Dio2 signaling axis governs cardiomyocyte thyroid hormone metabolism and hypertrophic growth
Source: Nat Commun. 2020 May 21;11:2551. doi: 10.1038/s41467-020-16345-y (PMC7242347; doi:10.1038/s41467-020-16345-y)
Supplement: Supplementary file 1 — Supplementary Information [file 41467_2020_16345_MOESM1_ESM.pdf]

SUPPLEMENTARY INFORMATION

FoxO1-Dio2 Signaling Axis Governs Cardiomyocyte Thyroid Hormone Metabolism and Hypertrophic Growth

Anwarul Ferdous[1], Zhao V. Wang[1], Yuxuan Luo[1], Dan L. Li[1], Xiang Luo[1],

Gabriele G. Schiattarella[1], Francisco Altam*irano*[1], Herman I. May[1],

Pavan K. Battiprolu[1], Annie Nguyen[1], Beverly A. Rothermel[1,2],

Sergio Lavandero[1,3], Thomas G. Gillette[1], Joseph A. Hill[1,2*]

[1] *Department of Internal Medicine (Cardiology Division)*
[2] *Department of Molecular Biology*
University of Texas Southwestern Medical Center, Dallas, TX 75390-8573,
[3] *Advanced Center for Chronic Diseases (ACCDiS) and Center for Molecular Studies of the Cell (CEMC), Faculty of Chemical & Pharmaceutical Sciences and Faculty of Medicine, Universidad de Chile, Santiago 8380492, Chile.*

[*]**Correspondence:**

Joseph A. Hill, M.D., Ph.D.
Division of Cardiology
UT Southwestern Medical Center
6000 Harry Hines Blvd, NB11.200
Dallas, Texas, 75390-8573
Tel: 1-214-648-1400
Fax: 1-214-648-1450
Twitter: @josephahill
joseph.hill@utsouthwestern.edu

This PDF file includes:

Supplementary Figures (1-8) and Figure Legends

Supplementary Table 1

Figures

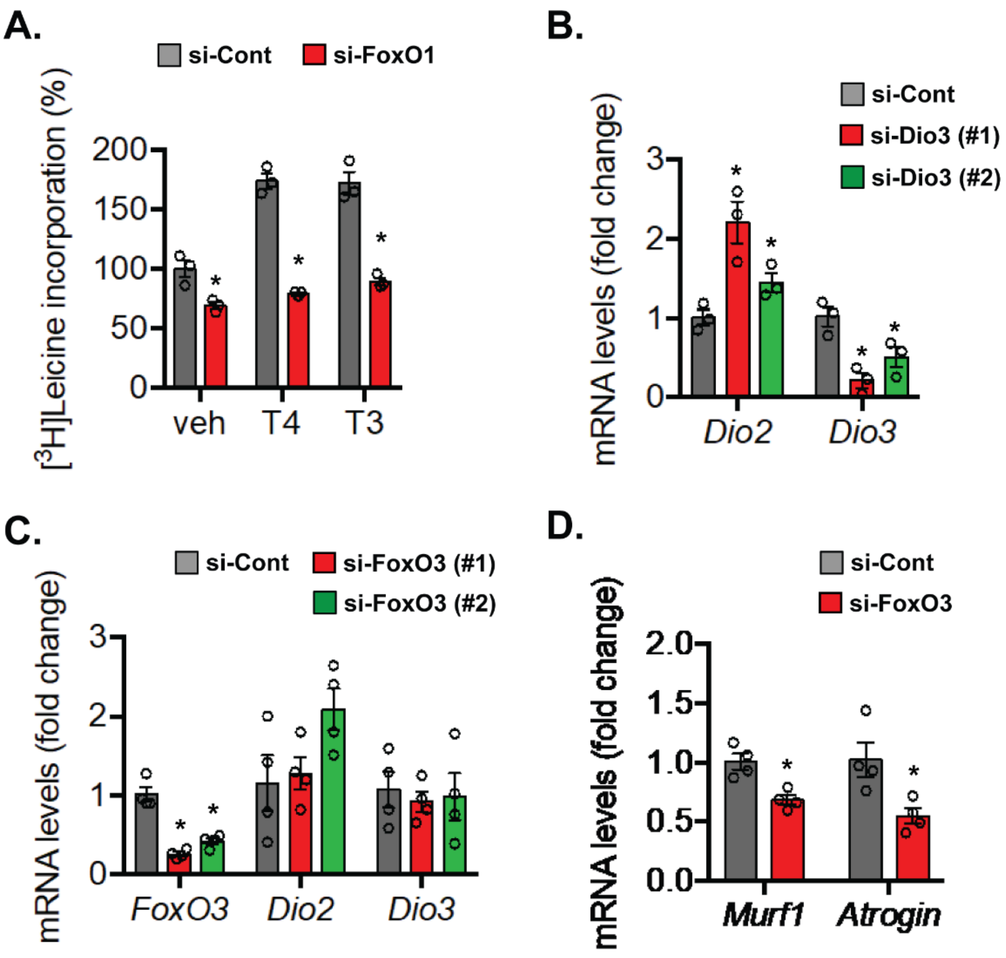

Supplementary Figure 1: FoxO1, but not FoxO3, is essential for TH-induced NRVM growth. (**A**) NRVMs were transfected with control or two sequence-independent *FoxO1*–specific siRNAs and evaluated for cell growth by assessing radiolabeled leucine incorporation into protein following 24h treatment with T4 or T3, where NRVM growth in the control (Cont) siRNA- and vehicle (Veh)-treated cells was set to 100%. (**B**) qRT-PCR analyses of mRNA levels of the indicated genes in NRVM transfected with control or two sequence-independent *Dio3*–specific siRNAs. (**C, D**) Selective knockdown of *FoxO3* in NRVM did not affect *Dio2* or *Dio3* (C), but significantly attenuated its target *Murf1* and *Atrogin* gene expression (D). In each panel, data are depicted as mean ± SEM (n=3 independent experiments). *p<0.05 vs control.

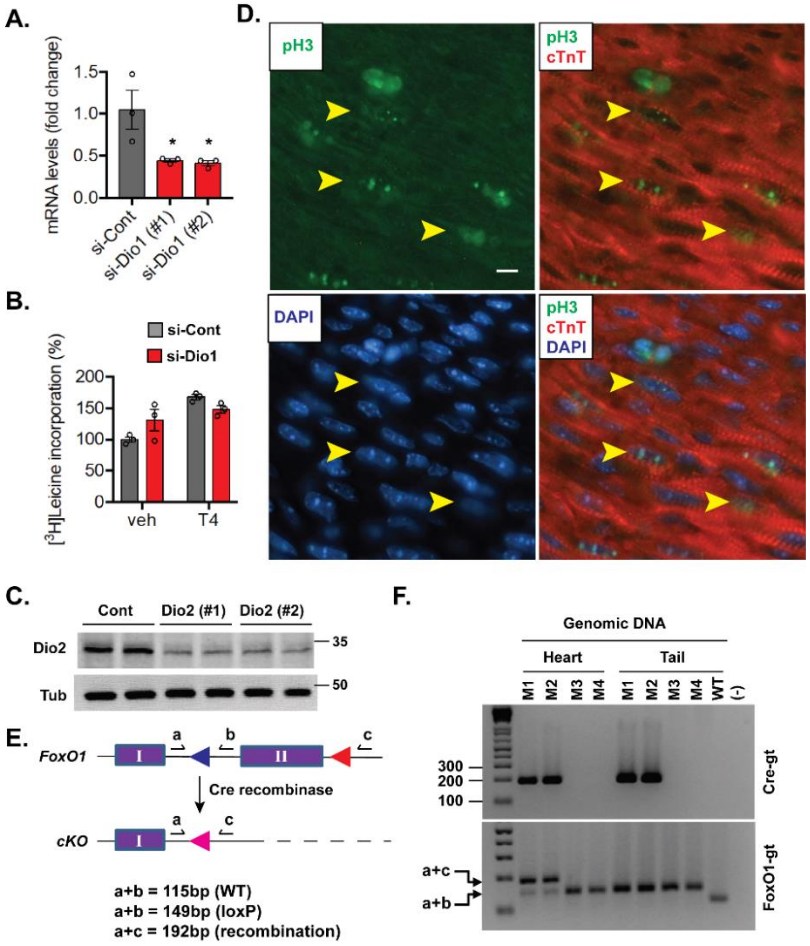

Supplementary Figure 2: FoxO1-Dio2 axis is essential for neonatal cardiomyocyte growth *in vitro* and *in vivo*. (**A, B**) qRT-PCR analyses of *Dio1* mRNA levels in NRVM transfected with control or two sequence-independent *Dio1*–specific siRNAs (A) and evaluated for cell growth following 24h (B) as described in Supplementary Figure 1A. Note that T4-induced NRVM growth was not affected in Dio1-deficient cells. In each panel, data are depicted as mean ± SEM (n=3 independent experiments), *$p<0.05$ vs control. (**C**) Immunoblot analyses of Dio2 levels in NRVM transfected with control or two sequence-independent *Dio2*–specific siRNAs. Tubulin (Tub) was used as loading control. (**D**) Co-immunostaining with anti-phospho-histone 3 [pH3 (green)] and anti-cTnT (red) antibodies of P3 WT heart. DAPI was used for nuclear staining (bar = 100μ). Amplified view of myocardium indicates proliferating cardiomyocytes (pH3 and cTnT positive nuclei) (arrowheads). (**E**) Top: schematic of *FoxO1* floxed allele depicting primer pairs (a-c) used to assess Cre-mediated recombination of loxP sites (triangle) spanning exon II. Bottom: lengths of expected PCR products in base pair (bp) with corresponding primer pairs and genotype are shown. (**F**) PCR analyses of genomic DNA (gDNA) from ventricle and tail of the indicated P3 mice (M1-M4) were used for genotyping (gt) of *Cre* (top) and *FoxO1* (bottom) genes. Note that robust Cre-mediated recombination was detected in Cre+ hearts only. PCR reactions without gDNA (-) and WT tail were used as control.

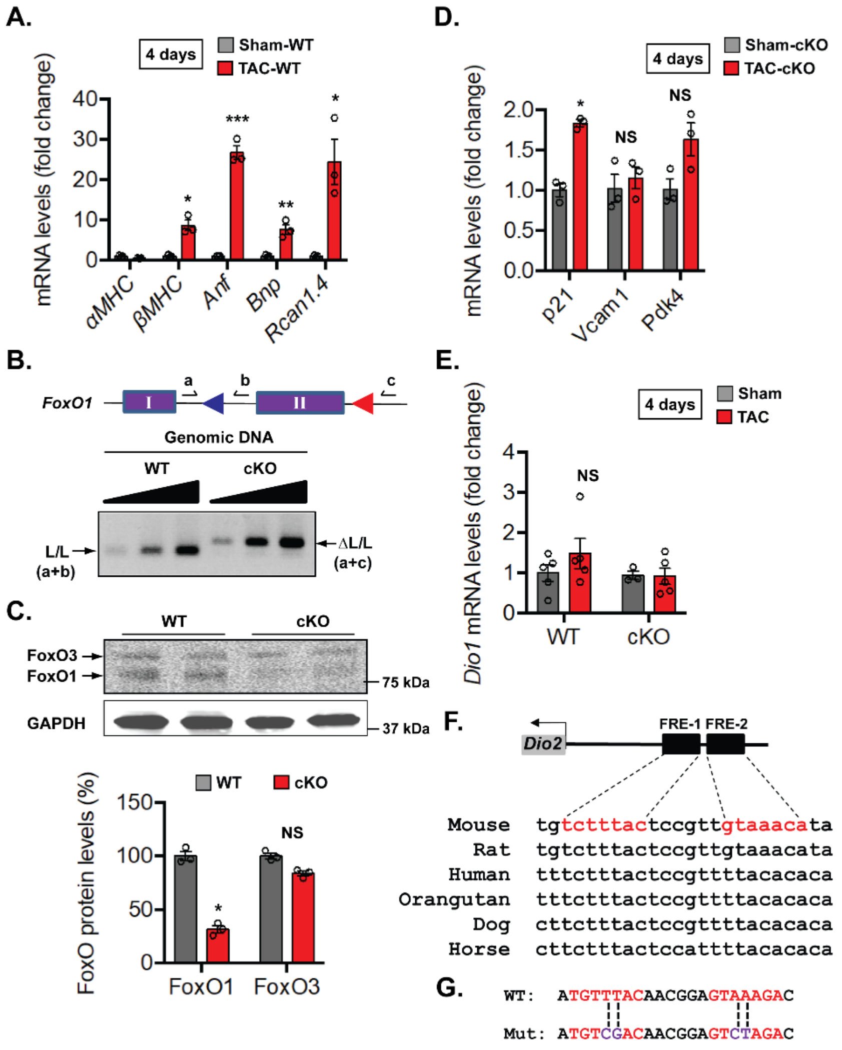

Supplementary Figure 3: Activation of FoxO1 and fetal gene program in early post-TAC heart. (**A**) qRT-PCR analyses of mRNA levels of the indicated fetal genes in 4 days TAC and sham LVs of *FoxO1-WT* (WT) mice (n = 3). (**B**) Top: schematic of *FoxO1* allele indicating primer pairs (a-c) used to assess Cre-mediated recombination of loxP sites (triangle) spanning exon II. Bottom: PCR analyses using increasing amounts of genomic DNA from ventricular cardiomyocytes of WT and *FoxO1-cKO* (cKO) mice demonstrate robust Cre-mediated recombination. (**C**) Immunoblotting (top) and quantitation (bottom) of FoxO1 and FoxO3 levels in LV lysates of WT and cKO hearts. GAPDH was used as loading control (n = 3). (**D, E**) qRT-PCR analyses of mRNA levels of the indicated genes in 4 days TAC and sham LVs of cKO (n = 6) (D) and WT (n = 10) and cKO (n = 8) (E) mice. (**F**) Schematic of *Dio2* promoter harboring two FoxO-responsive elements (FREs) and sequence alignment of conserved FREs in *Dio2* upstream promoter region of the indicated species are shown. (**G**) Nucleotide sequence of WT (red) and mutated (Mut) FREs. Mutated nucleotides are indicated in blue. In all panels, data are depicted as mean ± SEM. *p<0.05 vs sham; **p<0.01 vs sham and ***p<0.001 vs sham; NS, not statistically significant.

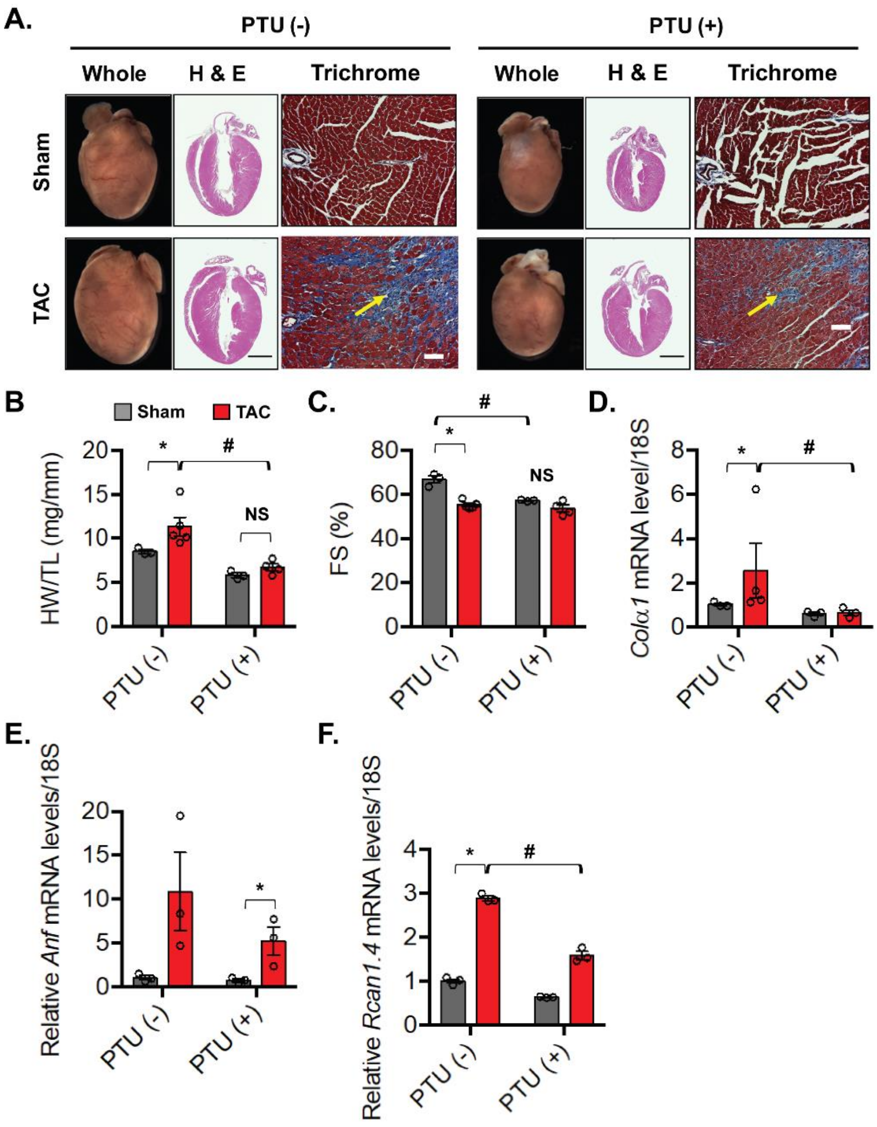

Supplementary Figure 4: Pharmacological inhibition of Dio2 activity attenuates pressure overload-induced cardiac remodeling and fibrosis. (**A**) Male mice fed normal [PTU(-)] or propylthiouracil (PTU)-containing [PTU(+)] chow were subjected to sham or TAC surgery. Whole mount (left), H&E (middle), and Masson trichrome staining (right) of representative 3-week sham and TAC hearts are shown. Note that TAC-induced fibrosis (arrows) and hypertrophic growth of the heart was blunted in PTU chow-fed mice. (**B**) Heart weight (HW)/tibia length (TL) ratios indicate robust cardiac hypertrophy in 3-week post-TAC normal chow-fed WT (n = 8) mice, which is attenuated in PTU chow-fed (n = 7) mice. (**C**) Percent of left ventricular fractional shortening (%FS) of 3-week sham- and TAC-operated hearts are shown. Note that TAC-induced marked contractile dysfunction in normal chow-fed (n = 8) mice was not observed in PTU chow-fed (n = 7) mice. (**D-F**) qRT-PCR analyses of mRNA levels of indicated genes in LVs of 3-week TAC- and sham-operated mice fed the indicated diet (n = 6). In all panels, data are depicted as mean ± SEM. **p*<0.05 vs sham; $^{\#}$*p*<0.01 vs PTU (-).

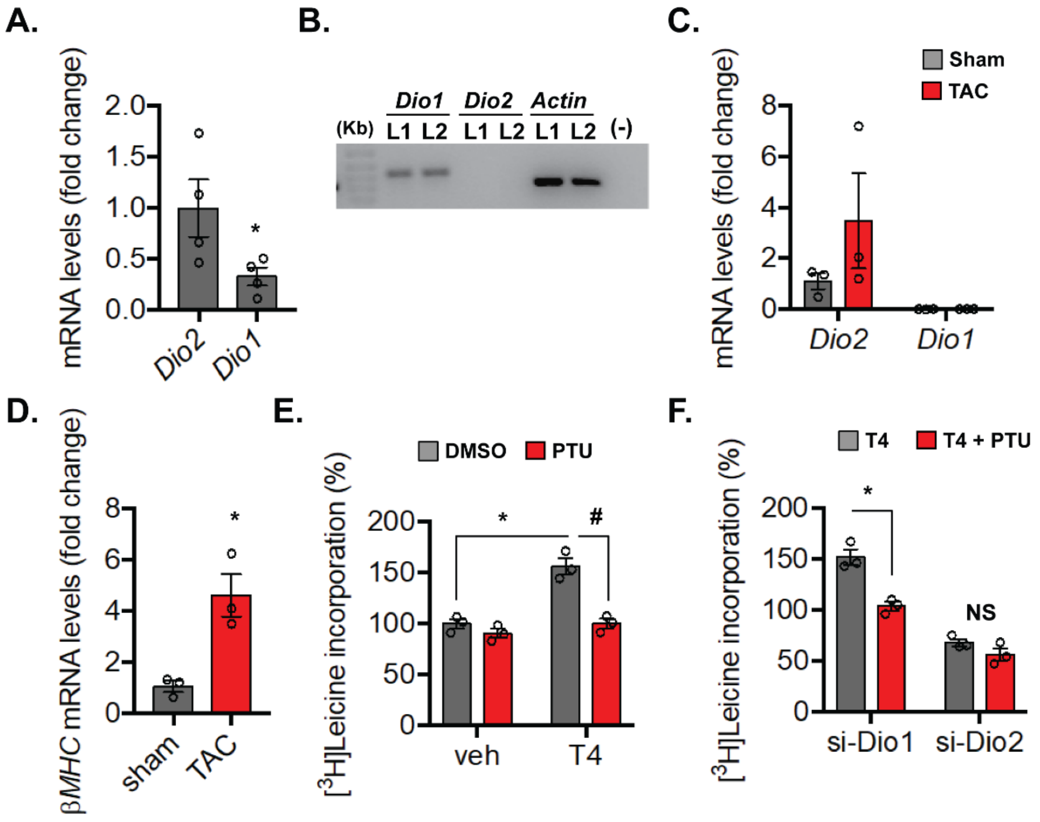

Supplementary Figure 5. PTU inhibits Dio2 activity, the major deiodinase enzyme in cardiomyocytes. (**A**) qRT-PCR analyses of mRNA levels of the indicated genes in LV of WT mice (n = 4), where relative *Dio2* expression was set to 1. (**B**) Semi-quantitative PCR analyses of mRNA abundance of the indicated genes in livers (L1 and L2) of WT mice. Note that *Dio2* and *Dio1* expression is higher in heart and liver, respectively. (**C, D**) qRT-PCR analyses of mRNA levels of the indicated genes in adult ventricular cardiomyocytes isolated from 3-week post-TAC and sham hearts (n = 3). (**E, F**) NRVMs were transfected with control (E) or indicated gene–specific siRNAs (F) and evaluated for cell growth following 36h of T4 and PTU treatment, where NRVM growth in the vehicle (Veh)- and DMSO-treated cells was set to 100% (n = 3 independent experiments). In all panels, data are depicted as mean ± SEM. *p<0.05 vs control/sham; $^{\#}p$<0.01 vs control; NS, not statistically significant.

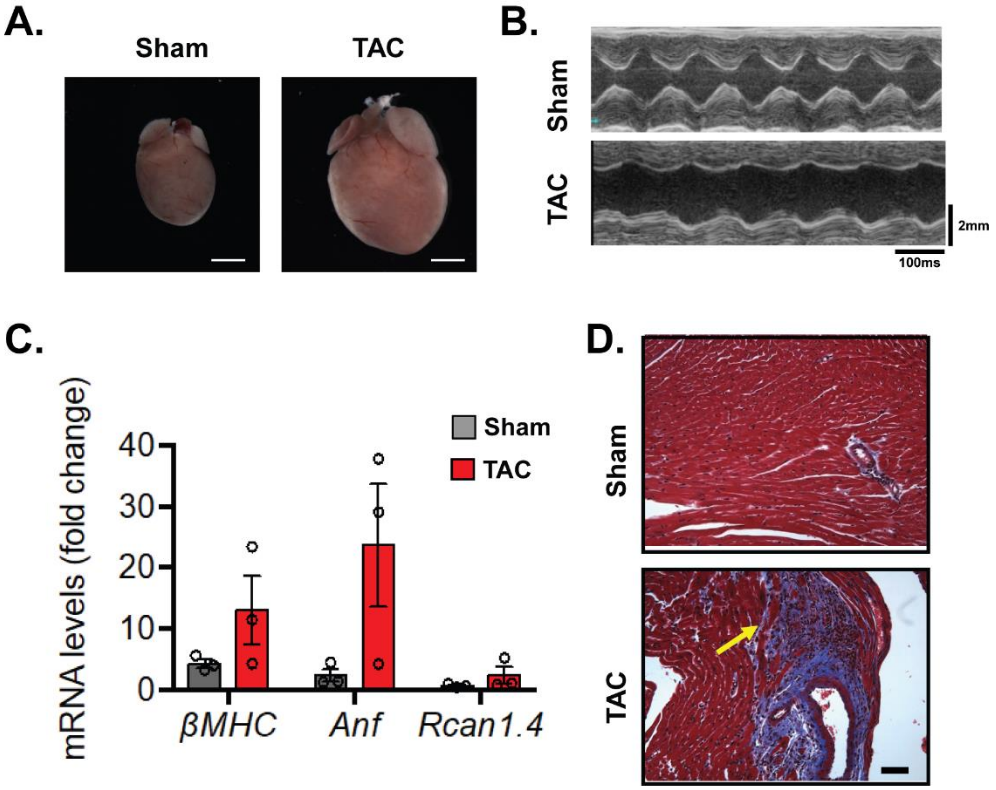

Supplementary Figure 6. TAC-induced cardiac hypertrophy in αMHC-Cre mice. (**A**) Whole mount of representative 3-week sham and TAC hearts are shown. (**B**) Representative M-mode tracings of 3-week sham- and TAC-operated hearts. (**C**) qRT-PCR analyses of mRNA levels of the indicated genes in 3-week TAC and sham (n = 3) LVs. Data are depicted as mean ± SEM. (**D**) Masson trichrome staining showing interstitial fibrosis (arrow) in TAC hearts.

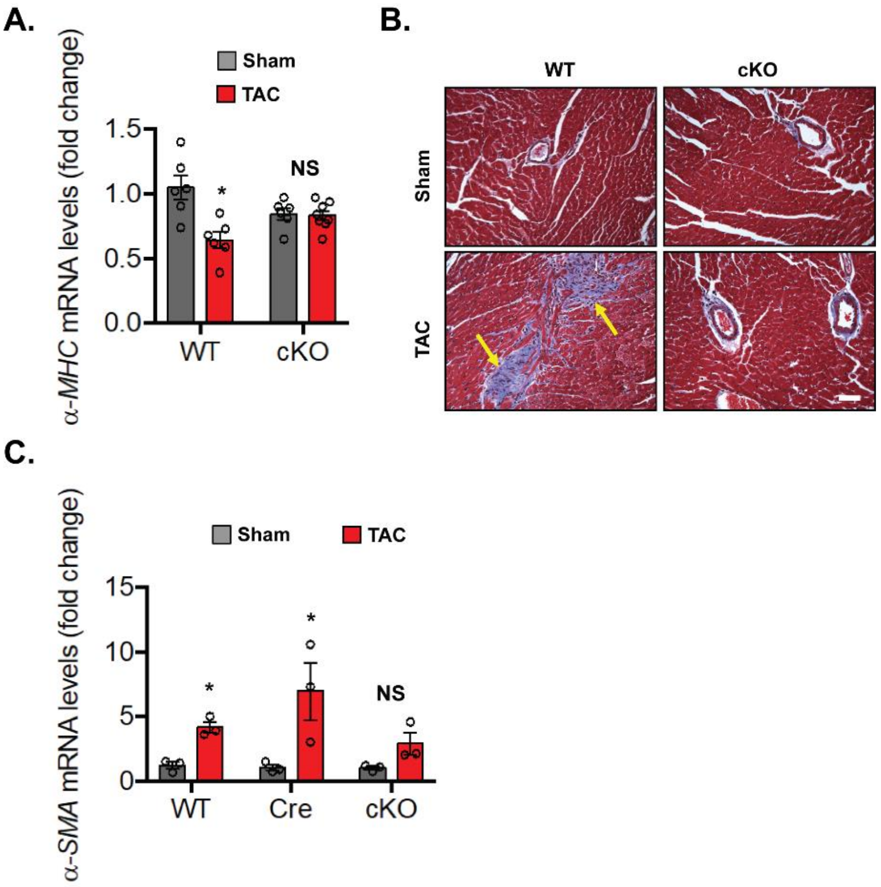

Supplementary Fig. 7: TAC-induced cardiac fibrosis and expression of fibroblast differentiation marker are blunted in *FoxO1-cKO* (cKO) hearts. (**A**) qRT-PCR analyses of mRNA levels of *α-MHC* in 3-week TAC and sham LVs of *FoxO1-WT* (WT, n = 12) and cKO (n = 13) mice. (**B**) Masson trichrome staining depicting marked increases in interstitial fibrosis (arrows) in 3-week TAC hearts of WT, but not cKO, mice. (**C**) qRT-PCR analyses of mRNA levels of *α-SMA* in 3-week TAC and sham LVs of WT (n = 6), Cre (n = 6) and cKO (n = 6) mice. In all panel, data are depicted as mean ± SEM. **p*<0.05 vs sham, NS, not statistically significant.

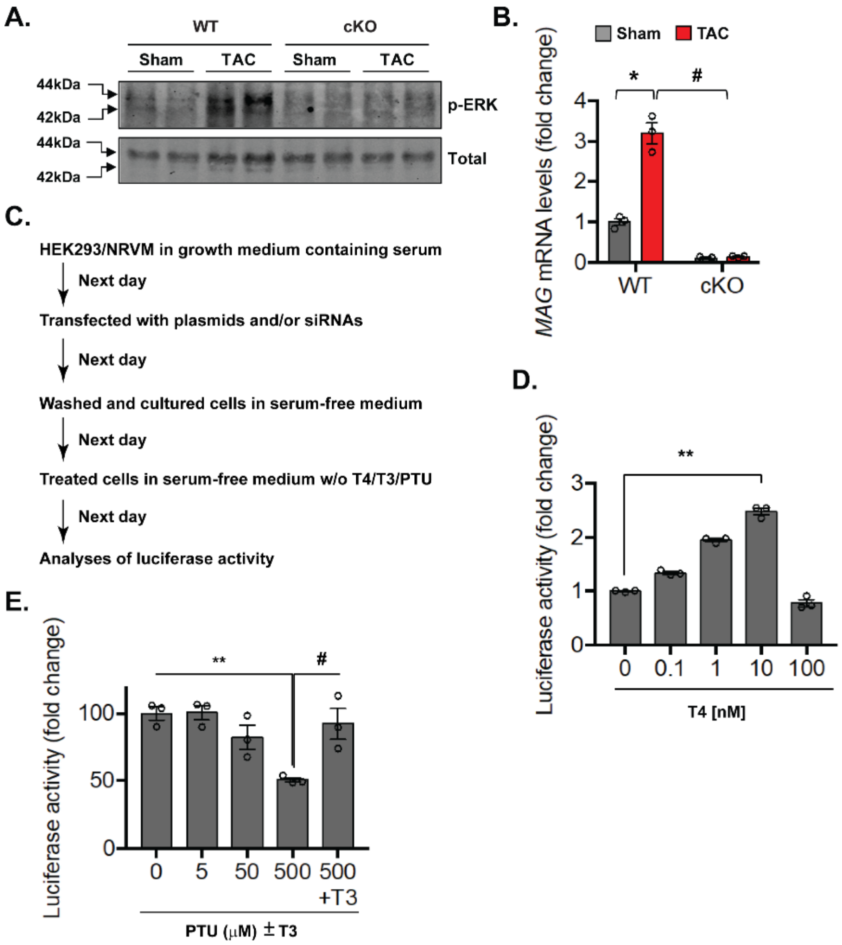

Supplementary Figure 8: FoxO1-Dio2 axis potentiates ERK and thyroid hormone receptor activity. (**A**) Immunoblot detection of total and phosphorylated ERK (p-ERK) levels in 3-week TAC and sham LVs of *FoxO1-WT* (WT) and *FoxO1-cKO* (cKO) mice (n = 3). (**B**) qRT-PCR analyses of mRNA levels of *Mag*, a downstream target of the thyroid hormone receptor (THR), in 3-week TAC and sham LVs of WT and cKO mice (n = 6). Note that *Mag* transcripts were undetectable in LVs of cKO mice. (**C**) Schematic of THR-responsive reporter assays in HEK293 and NRVM cells. HEK293 were transfected with reporter plasmid plus caFoxO1 and THRα1 (TRα1) expression plasmids alone or in combination. NRVMs were transfected with control or gene–specific siRNAs and 6h later, cells were washed and reporter plasmid was transfected. After 24 hours, both HEK293 and NRVM cells were washed and exposed to serum-free medium overnight to deplete endogenous TH. Next day, cells were washed with serum-free medium and treated with or without vehicle (0.05N NaOH), TH (T4 or T3) and PTU. Firefly luciferase activity was analyzed after 24 hours and normalized with Renilla activity. (**D**) Reporter activity in the presence of indicated concentrations of T4 in HEK293 cells co-transfected with reporter plasmid and expression vector of both caFoxO1 and TRα1. (**E**) Percent reporter activity in the presence of T4 and indicated concentrations of PTU in NRVM is shown. Reporter activity with no PTU was set to 100%. Note that addition of T3 (10nM) completely rescued the inhibitory effect of PTU (n = 3 independent experiments). In each panel, data are depicted as mean ± SEM. **p*<0.01 vs sham; ***p*<0.001 vs control; #*p*<0.01 vs WT/control,.

Supplementary Table 1. Primers used in this study

Gene	Primer	Reference
18s (mouse)	Taqman (SKU# 4333760T)	Ferdous et al., 2011
Vcam1 (mouse)	Taqman (Mm01320970_m1)	Ferdous et al., 2011
p21 (mouse)	Taqman (Mm01303209_m1)	Ferdous et al., 2011
CamKIIδ (mouse)	Taqman (Mm00499266_m1)	
18s (mouse)	AGGGTTCGATTCCGGAGAGG<br>CAACTTTAATATACGCTATTGG	
Pdk4 (mouse)	CGTTCCTTCACACCTTCACC<br>TGTTGGAGCAGTGGAGTACG	
Murf1 (mouse)	ACAACCTCTGCCGGAAGTGT<br>CGGAAACGACCTCCAGACAT	
βMHC (mouse)	AAGCAGCAGTTGGATGAGCG<br>CCTCGATGCGTGCCTGAAGC	
αMHC (mouse)	CGGAACAAGACAACCTCAAT<br>TGGCAATGATTTCATCCAGC	
Anf (mouse)	CTTCTTCCTCGTCTTGGCCT<br>CTGCTTCCTCAGTCTGCTCA	
Sma (mouse)	AGACCACCGCTCTTGTGTGT<br>GTCAGGATACCTCGCTTGCT	
Mag (mouse)	GCTACACTTCGTGCCTAC<br>AATTCATCTCCACAATCACTG	
Rcan1.4 (mouse)	CCCGTGAAAAAGCAGAATGC<br>TCCTTGTCATATGTTCTGAAGAGGG	
Klf15 (mouse)	GTTTCCCAAGAACCCAGCAGC<br>TCTGAGCGGGAAAACCTCCAG	

Colα1 (mouse)	CTCGTGGATTGCCTGGAACA<br>CCAACAGCACCATCGTTACC
Ctgf1 (mouse)	TGCACTTGCCTGGATGG<br>GGCAGTTGGCTCGCATCATA
Dio2 (mouse)	CTTCCTCCTAGATGCCTACAAAC<br>CGAGGCATAATTGTTACCTGATTC
Dio3 (mouse)	CCGCTCTCTGCTGCTTCAC<br>CGGATGCACAAGAAATCTAAAAGC
FoxO1 (mouse)	GCAGCCAGGCATCTCATAA<br>CCTACCATAGCCATTGCAGC
18s (rat)	AAACGGCTACCACATCCAAG<br>CCTCCAATGGATCCTCGTTA
Dio2 (rat)	CTTTCTCCTAGACGCCTACAAAC<br>GGCACAATTGTTACCTGCTTCAGG
Dio3 (rat)	GCGCTCCCTGCTGCTTCAC<br>CGGATGCACAAGAAATCTAAAAGC
Murf1 (rat)	GGTTCGGGGAGGAAGACAAA<br>TTGCTTCGAAACTGCGACTG
Atrogin (rat)	GCTTTGCAAACACTGCCACA<br>GGGGTGAAAGTGAGACGGAG
FoxO3 (rat)	TAAGCAAGCCGTGTACCGTG<br>TTGAGTGACGCAGGTCCGAA
FoxO1 (rat)	GCAAGACCAGTTCGTCGC<br>TTGAATTCTTCCAGCCCGCC
Anf (rat)	CTTCTTCCTCTTCCTGGCCT<br>TTCATCGGTCTGCTCGCTCA
BNP (rat)	TCCTTAATCTGTCGCCGCTG<br>AGGCGCTGTCTTGAGACCTA

Dio1 (mouse)	AAGAAGCTCACGCCACAGAT<br>GATCCTGCCCTCCTGTATCA
Dio1 (rat)	GCCTCTCAGGACAGAAGTGC<br>GTCAGCTGTGGAGGCAAAGT
BNP (mouse)	CATGGATCTCCTGAAGGTGC<br>CCTTCAAGAGCTGTCTCTGG
