# Supplementary material for: FoxO1–Dio2 signaling axis governs cardiomyocyte thyroid hormone metabolism and hypertrophic growth
Source: Nat Commun. 2020 May 21;11:2551. doi: 10.1038/s41467-020-16345-y (PMC7242347; doi:10.1038/s41467-020-16345-y)
Supplement: Supplementary file 3 — Reporting Summary [file 41467_2020_16345_MOESM3_ESM.pdf]

nature research

Corresponding author(s): NCOMMS-19-20354-T

Last updated by author(s): 2020/02/24

Reporting Summary

Nature Research wishes to improve the reproducibility of the work that we publish. This form provides structure for consistency and transparency in reporting. For further information on Nature Research policies, see Authors & Referees and the Editorial Policy Checklist .

Statistics

For all statistical analyses, confirm that the following items are present in the figure legend, table legend, main text, or Methods section.

n/a	Confirmed	
☐	☒	The exact sample size (*n*) for each experimental group/condition, given as a discrete number and unit of measurement
☐	☒	A statement on whether measurements were taken from distinct samples or whether the same sample was measured repeatedly
☐	☒	The statistical test(s) used AND whether they are one- or two-sided<br>*Only common tests should be described solely by name; describe more complex techniques in the Methods section.*
☒	☐	A description of all covariates tested
☐	☒	A description of any assumptions or corrections, such as tests of normality and adjustment for multiple comparisons
☐	☒	A full description of the statistical parameters including central tendency (e.g. means) or other basic estimates (e.g. regression coefficient) AND variation (e.g. standard deviation) or associated estimates of uncertainty (e.g. confidence intervals)
☐	☒	For null hypothesis testing, the test statistic (e.g. *F*, *t*, *r*) with confidence intervals, effect sizes, degrees of freedom and *P* value noted<br>*Give P values as exact values whenever suitable.*
☒	☐	For Bayesian analysis, information on the choice of priors and Markov chain Monte Carlo settings
☒	☐	For hierarchical and complex designs, identification of the appropriate level for tests and full reporting of outcomes
☒	☐	Estimates of effect sizes (e.g. Cohen's *d*, Pearson's *r*), indicating how they were calculated

Our web collection on statistics for biologists contains articles on many of the points above.

Software and code

Policy information about availability of computer code

Data collection	Sonos 5500 system, VisualSonics Vevo 2100 system; VevoStrain software (Visual Sonics Inc.) - LI-COR Odyssey 3.0 software - Zen Software (Zeiss) - Leica Application Suite (LAS);
Data analysis	GraphPad Prism sotware 8.0, Microsoft Excel, Image J software version 2.0 - Simple Western program (ProteinSimple) - LI-COR Odyssey 3.0 software - VevoStrain software (Visual Sonics Inc.), Abobe Illustrator CS3

For manuscripts utilizing custom algorithms or software that are central to the research but not yet described in published literature, software must be made available to editors/reviewers. We strongly encourage code deposition in a community repository (e.g. GitHub). See the Nature Research guidelines for submitting code & software for further information.

Data

Policy information about availability of data

All manuscripts must include a data availability statement. This statement should provide the following information, where applicable:

- Accession codes, unique identifiers, or web links for publicly available datasets
- A list of figures that have associated raw data
- A description of any restrictions on data availability

The data supporting the findings of this study are available within the paper and its Supplementary Information. Datasets generated and/or analyzed in the current study are provided as separate Source Data for Figs. 1a, 1b, 1d-f, 2a-b, 2d-f, 3a-f, 4b, 5b-d, 6a-f and 7a-e, as well as Supplementary Figs. 1a-d, 2a-c, 3a, 3c-d, 4b-f, 5a, 5c-f, 6c, 7a, 7c, and 8b, 8d, and 8e. Other source data related to the study are available from the corresponding author upon reasonable request

Field-specific reporting

Please select the one below that is the best fit for your research. If you are not sure, read the appropriate sections before making your selection.

- [x] Life sciences
- [ ] Behavioural & social sciences
- [ ] Ecological, evolutionary & environmental sciences

For a reference copy of the document with all sections, see nature.com/documents/nr-reporting-summary-flat.pdf

Life sciences study design

All studies must disclose on these points even when the disclosure is negative.

Sample size	No statistical analysis was used to predetermine sample size. Estimates were made based on our previous experience, experimental approach, availability and feasibility required to obtain statistically significant results.
Data exclusions	No data were excluded from the analyses.
Replication	Data reported in this manuscript were reproduced over numerous independent experiments with sufficient cells/animals per group to demonstrate statistical significance.
Randomization	Mice were randomly assigned to each experimental/control group.
Blinding	Investigators were blinded to the genotypes of the individual animals during the experiments and outcome assessments.

Reporting for specific materials, systems and methods

We require information from authors about some types of materials, experimental systems and methods used in many studies. Here, indicate whether each material, system or method listed is relevant to your study. If you are not sure if a list item applies to your research, read the appropriate section before selecting a response.

Materials & experimental systems

n/a	Involved in the study	
[ ]	[x]	Antibodies
[ ]	[x]	Eukaryotic cell lines
[x]	[ ]	Palaeontology
[ ]	[x]	Animals and other organisms
[x]	[ ]	Human research participants
[x]	[ ]	Clinical data

Methods

n/a	Involved in the study	
[x]	[ ]	ChIP-seq
[x]	[ ]	Flow cytometry
[x]	[ ]	MRI-based neuroimaging

Antibodies

Antibodies used	FoxO1 (#2880, Cell Signaling and ab39670; Abcam); Phospho-FoxO1 (#9464, Cell Signaling), ERK (#4695, Cell Signaling); Phospho-ERK (#4370, Cell Signaling); GAPDH (10R-G109a, Fitzgerald); Dio2 antibody (ab77481, Abcam); Tubulin (ab6046; Abcam).
Validation	All of antibodies were validated by vendors. We based specificity on their provided data sheets. For FoxO1 and Dio2 antibodies we performed the target validation using specific siRNAs.

Eukaryotic cell lines

Policy information about cell lines

Cell line source(s)	Adeno-X 293 cell line was purchased from Clontech (Adeno-X™ 293 Cell Line, cat# 632271);<br>COS-7 (ATCC; CRL-1651)
Authentication	Cell line was authenticated by the vendor.
Mycoplasma contamination	Mycoplasma detection was tested negative
Commonly misidentified lines<br>(See ICLAC register)	None

Animals and other organisms

Policy information about studies involving animals; ARRIVE guidelines recommended for reporting animal research

Laboratory animals	Stated in Methods under "Animals, Echocardiography and TAC".<br>FVB mice were used for wild-type (WT) studies. FoxO1 floxed mice were mice were crossed with the aMHC-Cre mouse model to generate mice with cardiomyocyte-specific deletion of FoxO1. All mice used in the cardiac hypertrophy experiments were adult male (8-10 week-old).
Wild animals	The study did not involve wild animals.
Field-collected samples	The study did not involve samples collected from the field.
Ethics oversight	All experiments involving animals conformed to the Guide for the Care and Use of Laboratory Animals published by the US National Institutes of Health (NIH Publication 8th edition, update 2011) and were approved by the Institutional Animal Care and Use Committee of the University of Texas Southwestern Medical Center.

Note that full information on the approval of the study protocol must also be provided in the manuscript.
